# Evaluating the triglyceride-glucose index as a biomarker for inflammatory bowel disease risk: insights from a UK Biobank prospective study

**DOI:** 10.3389/fimmu.2025.1548444

**Published:** 2025-08-27

**Authors:** Manli Zhang, Feng Hu, Ling Miao, Fan Li, Min Rao

**Affiliations:** Department of Hepatology and Gastroenterology, The First Hospital of Jilin University, Changchun, China

**Keywords:** inflammatory bowel disease, triglyceride-glucose index, UK Biobank, cohort study, biomarker

## Abstract

**Background:**

Inflammatory bowel disease (IBD), including ulcerative colitis (UC) and Crohn’s disease (CD), is a chronic inflammatory condition with rising global incidence. This study examines the association between the triglyceride-glucose (TyG) index, a marker of insulin resistance, and both the risk and prognosis of IBD.

**Methods:**

This study analyzed data from 327,089 participants in the UK Biobank. The TyG index was calculated based on fasting triglyceride and glucose levels, and participants were categorized into quartiles. Cox proportional hazards models, restricted cubic splines (RCS), subgroup analyses, and mediation analyses were employed to explore the association between TyG and the risk of UC and CD. Covariates were adjusted for age, sex, race, socioeconomic and lifestyle factors, physical activity, common chronic diseases, and polygenic risk scores. Multiple sensitivity analyses were conducted to ensure the robustness of the results.

**Results:**

An elevated TyG index was significantly associated with an increased risk of IBD. For each unit increase in TyG, the risk of UC increased by 14.3% (HR: 1.143, 95% CI: 1.053–1.241), while the risk of CD increased by 13.8% (HR: 1.138, 95% CI: 1.006–1.286). Participants in the highest TyG quartile had a 28.0% higher risk of CD (HR: 1.280, 95% CI: 1.050–1.560) and a 17.8% higher risk of UC (HR: 1.178, 95% CI: 1.028–1.351) compared to those in the lowest quartile. Mediation analysis revealed that the association between TyG and the incidence of CD and UC was mediated by multiple factors, including white blood cell count, mean corpuscular hemoglobin, C-reactive protein, total bilirubin, neutrophil count, reticulocyte percentage, and high-light scatter reticulocyte percentage. The proportion of mediation effects ranged from 1.44% to 30.97%.

**Conclusion:**

This study is the first to systematically investigate the relationship between the TyG index and both the risk and prognosis of IBD, using a large cohort from the UK Biobank. The findings reveal a significant positive association between the TyG index and the risk of developing UC and CD, suggesting that a higher TyG index may serve as an independent predictor of IBD risk.

## 1 Introduction

Inflammatory bowel disease (IBD), encompassing ulcerative colitis (UC) and Crohn’s disease (CD), represents a chronic inflammatory condition of the gastrointestinal tract. In recent years, the incidence of IBD has shown a rising trend globally, affecting millions of individuals. In Europe alone, the number of affected cases exceeds 3.2 million (1, 2). Although the precise etiology of IBD remains incompletely understood, current research suggests that it results from a multifactorial interplay involving genetic susceptibility, immune dysregulation, and dysbiosis of the gut microbiota (3, 4).

In recent years, the potential link between metabolic disturbances and IBD has gained increasing attention. A growing body of evidence suggests that metabolic syndrome and insulin resistance (IR) may play a pivotal role in the onset and progression of IBD (5, 6). Studies have demonstrated that the prevalence of metabolic syndrome is significantly higher in patients with IBD compared to the general population. Additionally, metabolic syndrome may elevate the risk of IBD development and disease activity (7). This bidirectional relationship indicates the involvement of shared pathophysiological mechanisms in the progression of both conditions.

Among the metabolic disturbances linked to IBD, insulin resistance, a core feature of metabolic syndrome, has garnered particular interest. Research has identified a significant state of insulin resistance in IBD patients, which is closely associated with disease activity and levels of inflammatory markers (8). Insulin resistance may not only increase the risk of developing IBD but could also influence disease prognosis (9). These findings provide important insights into the role of metabolic factors in the pathogenesis of IBD.

In this context, the Triglyceride-Glucose Index (TyG), calculated from fasting triglyceride and glucose levels, has emerged as a novel, simple, and effective marker for insulin resistance. In recent years, it has gained widespread application in the assessment of metabolic health (10, 11). Studies have shown that the TyG index not only serves as a tool for measuring insulin resistance but is also associated with the risk of several chronic conditions, including diabetes, hypertension, and cardiovascular diseases (12, 13). However, the relationship between the TyG index and the risk of IBD has not been thoroughly investigated.

Given the critical roles of insulin resistance and chronic inflammation in the pathogenesis of various diseases, we hypothesize that the TyG index may be potentially linked to the risk of developing IBD. To test this hypothesis, the present study aims to explore the association between the TyG index and the risk and prognosis of IBD by utilizing data from the large-scale UK Biobank cohort. We employed a variety of analytical methods, including Cox proportional hazards models, restricted cubic splines (RCS), subgroup analysis to assess interaction effects, and mediation analysis. This study will contribute to a deeper understanding of the complex relationship between metabolic disorders and IBD, offering valuable insights for the development of future intervention strategies and therapeutic approaches.

## 2 Methods

### 2.1 Data source

**Figure 1:**
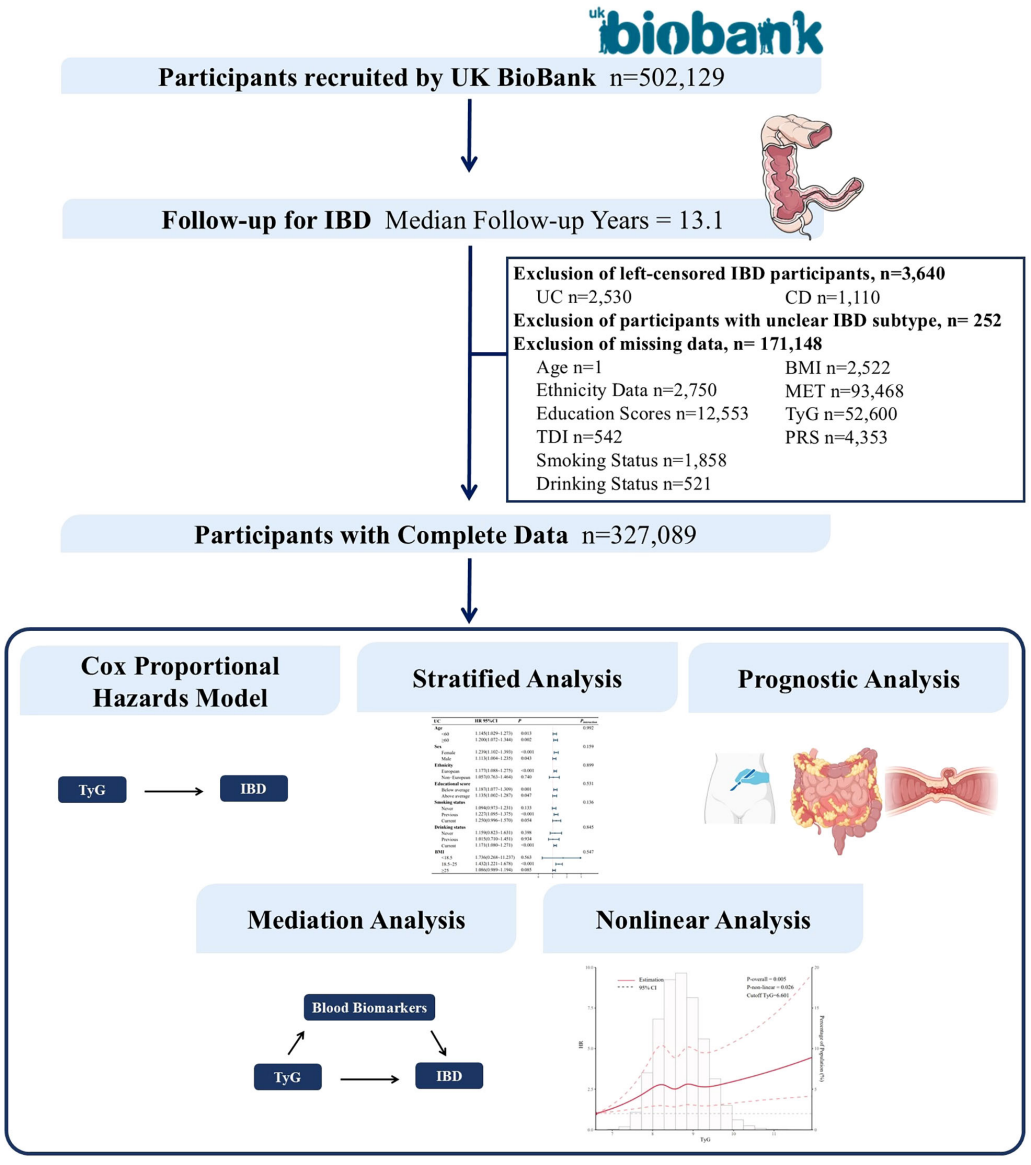
Flowchart of the study design. IBD, inflammatory bowel disease; TDI, Townsend Deprivation Index; BMI, body mass index.

The UK Biobank is a large-scale and detailed prospective study that recruited over 500,000 participants aged 40 to 69 years over a four-year period starting in 2006. This study has collected, and continues to follow up on, a wide range of phenotypic and multi-omics data, including questionnaire data, physical measurements, sample analyses, whole-genome genotyping data, and extensive health-related outcomes through longitudinal tracking. The UK Biobank obtained ethical approval from the North West Multi-centre Research Ethics Committee (REC reference: 11/NW/03820). All participants provided written informed consent for data collection, analysis, and linkage, and the study was conducted in accordance with the principles of the Declaration of Helsinki (14). Access to the UK Biobank data was granted under application ID 84347. This study was reviewed and approved by the Ethics Committee of the First Hospital of Jilin University (Approval No.: Lin Shen 2024-1263). Our dataset comprised information from 502,129 male and female participants. After excluding individuals with pre-existing IBD at recruitment (n = 3,640), those with undefined IBD subtypes (n = 252), and individuals with missing required data (n = 171,148), a total of 327,089 participants were included in the final analysis. Further details are provided in **Figure 1**.

### 2.2 Selection of variables, covariates, and outcomes

Peripheral venous blood samples were collected from all participants at baseline, and the collection procedures for the UK Biobank study were verified accordingly (15). The TyG index was calculated using the formula (16):


$$TyG=log_{e}\left(\frac{triglycerides\times fasting\;glucose}{2}\right)$$

where triglycerides and fasting glucose were both measured in mg/dL. Participants were then categorized into four groups (G1–G4) based on the quartiles of the TyG index (5.865, 8.311, 8.681, 9.078, 12.459).

All diseases, medication prescriptions, and participant deaths were recorded in the database for use and management. Participants were followed from the time of recruitment until the diagnosis of IBD, withdrawal from the study, death, or the last follow-up date, whichever occurred first. The diagnosis of IBD was based on hospital admission codes from the International Classification of Diseases, 10th and 9th editions (ICD-10, ICD-9). Specifically, “K50” or “555” indicated Crohn’s disease, while “K51” or “556” indicated ulcerative colitis.

In the models developed for this study, covariates were adjusted based on potential risk factors for IBD identified from previous research. The adjusted covariates included age, race (European, Asian, African, Chinese, mixed-race, and other), Townsend deprivation index, education level indicator, BMI, smoking status (current, former, never), alcohol consumption (current, former, never), metabolic equivalent of task (MET), hypertension, diabetes, coronary heart disease, malignancy, polygenic risk score for UC, and polygenic risk score for CD. Education attainment indicators were derived from data in the “Children and Youth” and “Adult Skills” domains, representing the “flow” and “stock” of educational disadvantage within a region. The Townsend Deprivation Index, introduced in 1987, is a tool used to measure socioeconomic deprivation, reflecting the level of material and social deprivation in a region. BMI was calculated from measured weight and height and categorized into underweight (BMI < 18.5 kg/m²), normal weight (18.5 kg/m² ≤ BMI < 25.0 kg/m²), and overweight/obesity (BMI ≥ 25.0 kg/m²) (17). Metabolic equivalent of task (MET) was used as an indicator to assess the intensity of participants’ daily physical activity based on a standardized questionnaire. It was calculated relative to resting metabolic rate (1 MET = 3.5 mL O_2_/kg/min) and incorporated activity type, duration, and frequency to estimate total energy expenditure. According to official recommendations, the weighting factors for walking, moderate activity, and vigorous activity were set at 3, 4, and 8, respectively. Relevant diseases such as hypertension and IBD complications (intestinal obstruction, intra-abdominal abscess, bowel perforation, fistula formation, toxic megacolon, Clostridium difficile infection, sepsis), IBD-related surgeries (small bowel resection, colectomy, anal fistula surgery), and all-cause mortality were extracted using the corresponding ICD-10 and ICD-9 codes. Detailed information on UK Biobank data extraction can be found in **Supplementary Table 1**. Polygenic risk score data were obtained from calculations published by Thompson et al., with the methodology described in a previous study (18).

The Dietary Inflammatory Index (DII) is a literature- and population-based tool designed to compare the inflammatory potential of diets across different populations (19). It has been widely applied in numerous studies (20, 21). In the UK Biobank, 29 eligible food or nutrient parameters were identified (**Supplementary Table 2**). Therefore, the DII used in this study was derived based on these 29 food and nutrient parameters. For the calculation of the energy-adjusted DII (E-DII), we followed the methodology outlined in previous research (22). First, food and nutrient intake was adjusted for total energy intake using the density method (nutrient or food intake/total energy intake × 1000 kcal). Then, the steps for DII calculation were repeated to obtain the E-DII.

### 2.3 Statistical methods and software

All analyses were pre-specified according to the Strengthening the Reporting of Observational Studies in Epidemiology (STROBE) guidelines prior to data inspection (23), as detailed in **Supplementary Table 3**. For the baseline characteristics, continuous variables were presented as means (± standard deviation), and statistical differences were assessed using the Student’s t-test. Categorical variables were reported as counts (percentages) and compared using Pearson’s Chi-squared test. In this study, we not only analyzed TyG as a continuous variable but also divided it into four groups for further analysis. A Cox proportional hazards model was employed to investigate the association between TyG and the incidence of IBD subtypes (UC, CD).

We applied three models to progressively adjust for covariates. Model 1 was adjusted for sex, age, race, Townsend deprivation index, and education score. Model 2 was further adjusted for smoking, alcohol consumption, BMI, and MET based on Model 1. Model 3 was additionally adjusted for hypertension, diabetes, coronary heart disease, malignancy, polygenic risk score for UC, and polygenic risk score for CD based on Model 2. The proportional hazards (PH) assumption for the Cox models was tested using the Schoenfeld residuals method, and all models met the PH assumption. Additionally, we performed subgroup analyses based on age, sex, race, education score, smoking status, alcohol consumption, and BMI to examine potential interactions between TyG and these variables.

We further employed RCS to assess potential nonlinear dose-response relationships between TyG and IBD. Beyond the etiological exploration, we also used the Cox proportional hazards model to investigate the effect of TyG on various IBD complications, IBD-related surgeries, and all-cause mortality in both the UC and CD populations.

Finally, multiple sensitivity analyses were conducted. First, E-DII was added to the model to account for potential dietary influences on the conclusions. Second, IBD cases diagnosed within 1 and 3 years after baseline assessment, as well as those diagnosed before the first follow-up, were excluded to further clarify the long-term impact of baseline TyG levels. Third, participants with cardiovascular disease or cancer were excluded. Fourth, multiple imputation was performed under the assumption that missing values were completely missing at random. The multiple imputation method was conducted using the Fully Conditional Specification (FCS) approach to generate five imputed datasets. The imputation model incorporated all variables used in the analyses, with a maximum of 40 iterations to ensure model convergence. Following imputation, statistical analyses were performed separately on each imputed dataset, and the final results were combined using Rubin’s rules.

All statistical analyses and plots were performed using R Project for Statistical Computing (version 4.3.3). All statistical tests were two-sided, and a p-value < 0.05 was considered statistically significant.

## 3 Results

### 3.1 Baseline characteristics

The baseline characteristics of participants, stratified by IBD subtype, are presented in **Supplementary Table 4**. Among the participants included in the study, females accounted for a higher proportion (52%), and the average age of participants was 56.3 ± 8.1 years. In the group of CD patients, the proportion of females was higher, with their average age being 0.53 years older than that of the non-IBD participants. Compared to non-IBD participants, CD patients tended to have higher education scores, higher Townsend Deprivation Index (TDI) scores, more current and past smoking habits, higher alcohol consumption, lower BMI, and higher TyG scores. In patients with UC, the proportion of males was higher, and their average age was 0.9 year older than that of non-IBD participants. Compared to non-IBD participants, UC patients generally had higher education scores, higher TDI scores, a greater proportion of past smokers, less alcohol consumption, higher BMI, and higher TyG scores. A total of 2,367 IBD patients were included in this study, comprising 740 patients with CD and 1,627 patients with UC. Baseline data stratified by TyG scores can be found in **Supplementary Table 5**.

### 3.2 Association between TyG and IBD subtypes

Cox proportional hazards model (**Table 1**) showed that an elevated TyG index was associated with an increased risk of developing IBD. In Model 3, continuously increasing TyG was significantly associated with the incidence of CD (HR, 95% CI: 1.138 [1.006–1.286], P = 0.040). Compared with the lowest TyG group (G1), the highest TyG group (G4) had a 28.0% higher risk of developing CD (HR, 95% CI: 1.280 [1.050–1.560], P = 0.015). Similarly, in Model 3, continuously increasing TyG was significantly associated with UC (HR, 95% CI: 1.143 [1.053–1.241], P = 0.001). Compared with G1, the G3 group had a 25.5% increased risk of UC (HR, 95% CI: 1.255 [1.101–1.432], P = 0.001), while the G4 group had a 17.8% increased risk (HR, 95% CI: 1.178 [1.028–1.351], P = 0.019).

**Table 1:** Cox regression results for continuous, categorical, and trend variables.

Outcomes	Model	Continuous		G1	G2		G3		G4		P for trend
		HR 95%CI	P		HR 95%CI	P	HR 95%CI	P	HR 95%CI	P	
UC	Model1	1.186(1.098~1.280)	<0.001	Ref.	1.068(0.937~1.217)	0.324	1.251(1.102~1.420)	0.001	1.231(1.083~1.399)	0.001	<0.001
	Model2	1.169(1.077~1.269)	<0.001	Ref.	1.070(0.937~1.221)	0.319	1.249(1.096~1.424)	0.001	1.204(1.051~1.379)	0.007	0.001
	Model3	1.143(1.053~1.241)	0.001	Ref.	1.078(0.943~1.232)	0.269	1.255(1.101~1.432)	0.001	1.178(1.028~1.351)	0.019	0.005
CD	Model1	1.207(1.076~1.353)	0.001	Ref.	1.030(0.848~1.250)	0.765	1.169(0.967~1.414)	0.107	1.379(1.145~1.662)	0.001	<0.001
	Model2	1.170(1.035~1.323)	0.012	Ref.	1.014(0.833~1.233)	0.893	1.134(0.933~1.379)	0.207	1.327(1.089~1.616)	0.005	0.002
	Model3	1.138(1.006~1.286)	0.040	Ref.	1.003(0.824~1.221)	0.977	1.127(0.926~1.371)	0.234	1.280(1.050~1.560)	0.015	0.006

The statistical models were adjusted in a stepwise manner. Model 1 accounted for sex, age, race, Townsend deprivation index, and education score. Model 2 was further adjusted for smoking status, alcohol consumption, BMI, and MET based on Model 1. Model 3 incorporated additional adjustments for hypertension, diabetes, coronary heart disease, malignancy, polygenic risk score for UC, and polygenic risk score for CD on top of Model 2. TyG was categorized into quartiles, with effect sizes representing comparisons with the first quartile (G1). Ptrend was used to assess the trend test. Abbreviations: HR, hazard ratio; CI, confidence interval; CD, Crohn’s disease; UC, ulcerative colitis.

**Figure 2:**
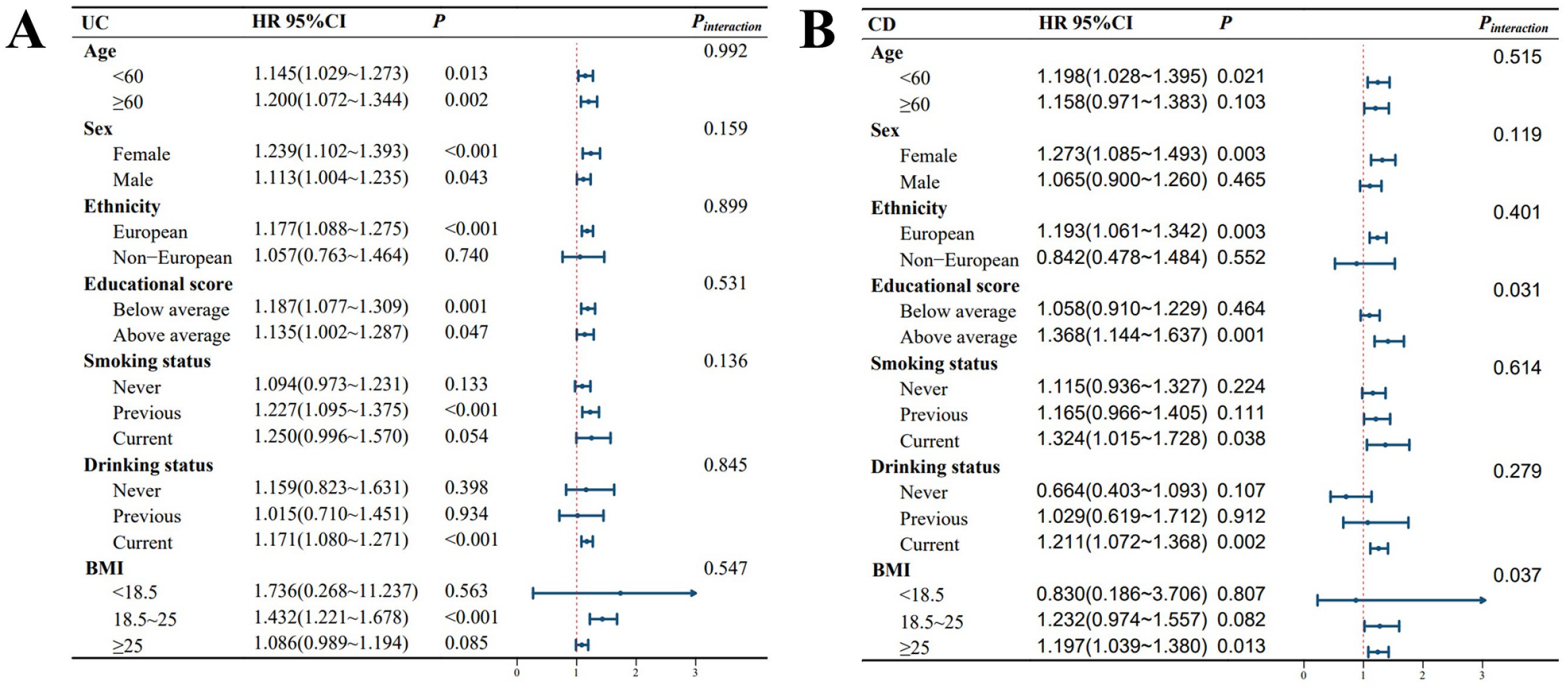
Forest plot of stratified analyses. P-interaction represents the P value for the significance of the interaction effect. The average education score was 14.31. Abbreviations: BMI, body mass index; HR, hazard ratio; CI, confidence interval. **(A)** presents the TyG-stratified analysis for UC, while **(B)** illustrates the TyG-stratified analysis for CD. All models used in the analysis were adjusted for the following covariates: sex, age, race, Townsend deprivation index, education score, smoking status, alcohol consumption, BMI, MET, hypertension, diabetes, coronary heart disease, malignancy, polygenic risk score for UC, and polygenic risk score for CD.

As shown in **Figure 2**, after adjusting for covariates, the impact of TyG on UC incidence was more pronounced among former smokers, current alcohol consumers, and individuals with normal weight (BMI between 18.5 and 25). For CD, after covariate adjustment, individuals under 60 years old, females, those with lower education levels, current smokers, current alcohol consumers, and those who were overweight or obese (BMI >25) were more susceptible to the influence of TyG on CD development. Additionally, a significant interaction between TyG and BMI was observed in relation to CD risk.

### 3.3 Study on mediators of the association between TyG and IBD

As shown in **Table 2**, mediation analysis indicated that the association between TyG and CD incidence was mediated by multiple factors, including white blood cell count, mean corpuscular hemoglobin, C-reactive protein (CRP), and total bilirubin. The top mediating factor was CRP, with a mediation effect proportion of 28.13% (P < 0.001). Similarly, the association between TyG and UC incidence was mediated by several factors, including white blood cell count, neutrophil count, reticulocyte percentage, and high-light scatter reticulocyte percentage. The top mediating factor was neutrophil count, with a mediation effect proportion of 30.97% (P < 0.001).

**Table 2:** Mediation analysis results for the effect of TyG on CD or UC.

Pathway	Total effects	P	Indirect effects	P	Mediated proportion (%)
	HR 95%CI		HR 95%CI		
TyG -> WBC -> CD	1.138 (1.012~1.280)	0.030	1.009 (1.002~1.016)	0.013	6.84%
TyG -> MCH -> CD	1.130 (1.005~1.271)	0.041	1.016 (1.012~1.020)	<0.001	13.20%
TyG -> CRP -> CD	1.137 (1.013~1.276)	0.029	1.037 (1.018~1.056)	<0.001	28.13%
TyG -> TB -> CD	1.135 (1.010~1.276)	0.034	1.026 (1.001~1.051)	0.038	20.05%
TyG -> WBC -> UC	1.157 (1.069~1.251)	<0.001	1.011 (1.004~1.018)	0.003	7.21%
TyG -> MCH -> UC	1.165 (1.077~1.260)	<0.001	1.003 (1.001~1.004)	<0.001	1.87%
TyG -> NEU -> UC	1.127 (1.042~1.220)	0.003	1.038 (1.034~1.042)	<0.001	30.97%
TyG -> RET% -> UC	1.166 (1.076~1.263)	<0.001	1.002 (1.001~1.004)	0.005	1.44%
TyG -> HLR% -> UC	1.164 (1.075~1.261)	<0.001	1.003 (1.001~1.005)	0.001	2.21%

All models used in the mediation analysis were adjusted for the following covariates: sex, age, race, Townsend deprivation index, education score, smoking status, alcohol consumption, BMI, MET, hypertension, diabetes, coronary heart disease, malignancy, polygenic risk score for UC, and polygenic risk score for CD. Abbreviations: TyG, triglyceride-glucose index; CD, Crohn’s disease; UC, ulcerative colitis; HR, hazard ratio; CI, confidence interval; WBC, white blood cell count; MCH, mean corpuscular hemoglobin; CRP, C-reactive protein; TB, total bilirubin; NEU, neutrophil count; RET%, reticulocyte percentage; HLR%, high-light scatter reticulocyte percentage.

### 3.4 Nonlinear analysis of TyG and IBD

**Figure 3:**
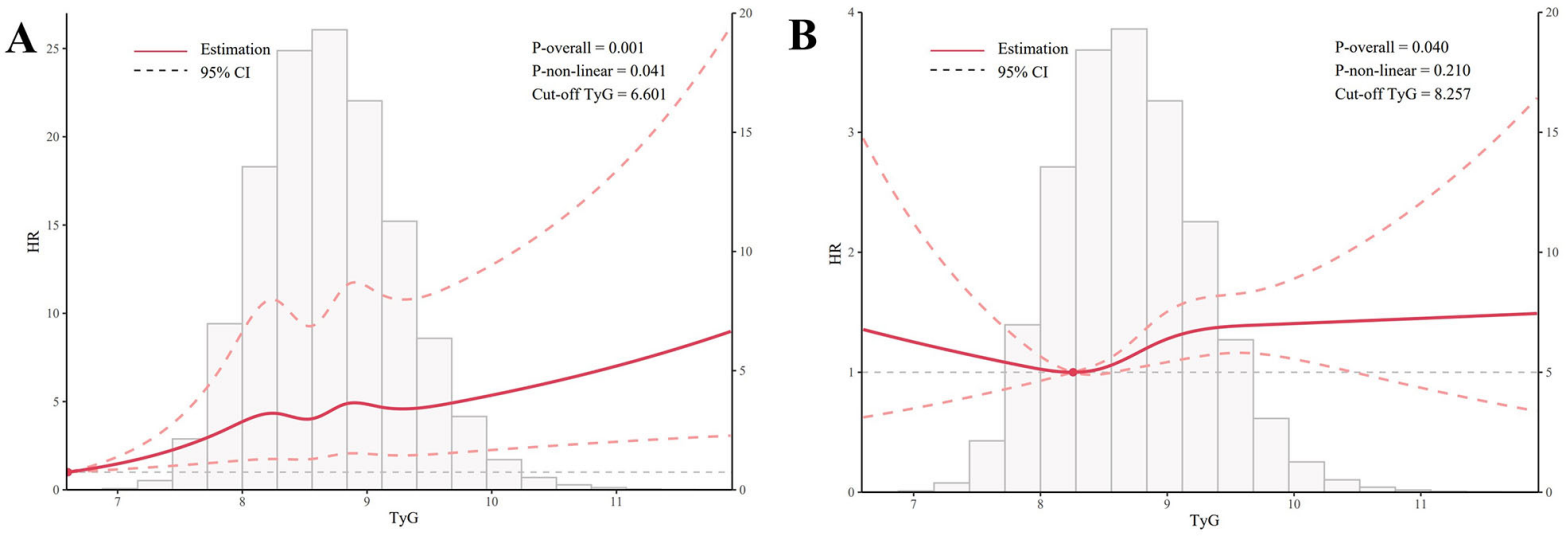
Graphical representation of non-linear relationships (Restricted Cubic Spline). **(A)** illustrates the nonlinear relationship between TyG and UC, while **(B)** depicts the nonlinear relationship between TyG and CD. All models used in the analysis were adjusted for the following covariates: sex, age, race, Townsend deprivation index, education score, smoking status, alcohol consumption, BMI, MET, hypertension, diabetes, coronary heart disease, malignancy, polygenic risk score for UC, and polygenic risk score for CD.

As shown in **Figure 3**, a nonlinear relationship was observed between the TyG index and UC incidence. The pattern was characterized by a sharp initial increase (TyG between 6.6 and 8.2), followed by a plateau phase (TyG between 8.2 and 9.2), and then another increase when TyG exceeded 9.2 (P for nonlinearity = 60.041). Although no significant nonlinear effect was observed between the TyG index and CD incidence (P = 0.210), the trend in the figure suggests an initial increase (TyG between 8.2 and 9.2), followed by a plateau phase when TyG exceeded 9.2.

### 3.5 Association of TyG with prognosis of IBD

We also analyzed the prognostic factors of UC and CD, including complications, infections, IBD-related surgeries, and all-cause mortality, using the TyG index. In the fully adjusted Cox regression model, a higher TyG index was associated with an increased risk of intestinal obstruction complications in CD patients (HR, 95% CI: 1.298(1.030~1.637); P = 0.027). Additionally, a higher TyG index was linked to a higher incidence of fistula complications in CD patients (HR, 95% CI: 1.398(1.015~1.925); P = 0.040) and an increased likelihood of IBD-related surgeries in CD patients (HR, 95% CI: 1.812(1.505~2.183); P < 0.001). No significant associations were found between the TyG index and other prognostic indicators in IBD patients (**Table 3**).

**Table 3:** Impact of TyG on the Prognosis of UC and CD.

IBD Subtypes	Complication	Sample size	Case	HR 95%CI	P
CD	Abscess	1707	58	1.549 (0.991~2.421)	0.055
	Obstruction	1684	226	1.298 (1.030~1.637)	0.027
	Fistula	1674	121	1.398 (1.015~1.925)	0.040
	All-cause Mortality	1739	213	1.069 (0.833~1.371)	0.601
	IBD-related Surgery	1646	351	1.812 (1.505~2.183)	<0.001
UC	Abscess	3733	48	0.764 (0.450~1.296)	0.317
	Obstruction	3713	158	1.024 (0.770~1.361)	0.873
	Fistula	3705	88	0.986 (0.669~1.453)	0.942
	Clostridium difficile infection	3738	32	1.584 (0.855~2.936)	0.144
	All-cause Mortality	3752	393	0.960 (0.803~1.147)	0.653
	IBD-related Surgery	3662	372	1.158 (0.959~1.397)	0.127

All models used in the analysis were adjusted for the following covariates: sex, age, race, Townsend deprivation index, education score, smoking status, alcohol consumption, BMI, MET, hypertension, diabetes, coronary heart disease, malignancy, polygenic risk score for UC, and polygenic risk score for CD. The analysis included all IBD patients from the UK Biobank database. However, due to the limited number of cases (fewer than 30), perforation, toxic megacolon, and Clostridium difficile infection (CD only) were not analyzed. Abbreviations: CD, Crohn’s disease; UC, ulcerative colitis; IBD, inflammatory bowel disease.

### 3.6 Sensitivity analysis

In the sensitivity analysis, after adding E-DII to the model, the conclusions remained consistent with previous findings (**Supplementary Table 6**), indicating that the effect of TyG was independent of individual dietary differences. Similarly, after excluding IBD cases diagnosed within 1 and 3 years after baseline assessment, as well as those diagnosed before the first follow-up, and after removing participants with cardiovascular disease or cancer, the conclusions remained unchanged (**Supplementary Tables 7–8**). Finally, after performing multiple imputations for missing covariates, the previously established conclusions remained robust (**Supplementary Table 9**).

## 4 Discussion

This study, based on the large-scale cohort data from the UK Biobank, is the first to systematically investigate the relationship between the TyG index and the risk and prognosis of IBD. It reveals a significant association between the TyG index and IBD, providing crucial evidence for the link between metabolic health and the pathogenesis of IBD. The findings indicate that, after adjusting for relevant covariates, the TyG index is positively associated with the risk of UC and CD. Specifically, for each 1-unit increase in the TyG index, the risk of UC and CD increases by 14.3% and 13.8%, respectively. This relationship was consistently observed not only in the analysis of continuous variables but also in the quartile and trend analyses, highlighting the potential value of the TyG index as a tool for assessing IBD risk.

The TyG index, calculated from fasting glucose and triglyceride levels, is commonly regarded as a biomarker of insulin resistance (24, 25). Our findings align with the recent research trends regarding the relationship between insulin resistance and IBD. A large cohort study reported an association between insulin resistance and an increased risk of IBD (9), which supports the context of our study. Moreover, the TyG index, as a simple and effective marker, has demonstrated significant utility in predicting and assessing MetS (26). Relevant studies suggest that MetS and IBD share similar pathophysiological characteristics, including inflammation, adipose tissue metabolism dysregulation, and immune response disorders (27, 28). Our research further extends this understanding, suggesting that even in individuals who do not meet the diagnostic criteria for MetS, subtle metabolic changes, as reflected by an elevated TyG index, may increase the risk of developing IBD. This finding is also of significant importance for the early prevention and risk management of IBD (29).

Some fundamental studies have indicated that insulin resistance is closely associated with an increase in the release of inflammatory factors, such as TNF-α and IL-6. It is believed that insulin resistance may alter gene expression within the NF-κB signaling pathway, leading to the upregulation of pro-inflammatory genes, thereby promoting a robust inflammatory response and immune activation (30). Furthermore, the enhanced release of inflammatory factors directly affects the inflammatory status of the intestinal mucosa, impacting the metabolism and function of intestinal epithelial cells, resulting in compromised intestinal barrier function and increased intestinal permeability, which may exacerbate the development of IBD (31). Moreover, chronic inflammation in IBD patients has been shown to exacerbate insulin resistance, creating a vicious cycle (32).

The mediation analysis in our study elucidated potential biological mechanisms underlying the association between the TyG and IBD. For CD, CRP exhibited the strongest mediating effect (28.13%, P < 0.001), highlighting the central role of systemic inflammation in the TyG-CD relationship, while white blood cell count, mean corpuscular hemoglobin, and total bilirubin mediation suggested the involvement of immune regulation, erythrocyte function, and oxidative balance in the pathophysiological link between TyG and CD (33, 34). For UC, neutrophil count emerged as the most significant mediator (30.97%, P < 0.001), aligning closely with the hallmark features of intestinal inflammation in UC, and the mediating roles of reticulocyte-related parameters indicated that erythropoietic abnormalities might represent another important pathway through which TyG influences UC pathogenesis. Notably, white blood cell count mediated associations of TyG with both CD and UC, suggesting immune system activation as a common pathway connecting insulin resistance to IBD (35), while the differences in primary mediators between CD and UC reflected subtype-specific pathological mechanisms. These findings not only deepen our understanding of the TyG-IBD association but also reveal a complex interaction network between metabolic dysregulation and intestinal inflammation, supporting the clinical value of monitoring and intervening in metabolic abnormalities among high-risk IBD populations, while simultaneously establishing a theoretical foundation for developing novel therapeutic strategies targeting these mediating pathways (32, 36).

This stratified analysis revealed that older age, female gender, and higher BMI populations exhibit a stronger correlation between UC and CD incidence and the TyG index. Moreover, a significant interaction was observed between the TyG index and gender in the onset of UC and CD. The following sections will elucidate the potential mechanisms underlying these associations. First, with increasing age, insulin sensitivity declines and metabolic function deteriorates. Older individuals are more prone to insulin resistance, which is closely associated with an elevated TyG index (37). Concurrently, immune function also decreases with age, potentially increasing susceptibility to IBD. Therefore, the impact of the TyG index on UC and CD is more pronounced in the elderly population. In addition, metabolic and immune responses differ between males and females. Estrogen plays a regulatory role in insulin sensitivity and lipid metabolism, potentially influencing the TyG index. Estrogen can enhance insulin signaling by activating the phosphoinositide 3-kinase (PI3K) and protein kinase B (Akt) pathways, which in turn promotes the phosphorylation of insulin receptor substrates (IRS) (38). Moreover, estrogen can activate AMP-activated protein kinase (AMPK), thereby increasing fatty acid oxidation and inhibiting the expression of lipogenic genes such as fatty acid synthase (FAS) and acetyl-CoA carboxylase (ACC) (39). Estrogen also affects the gut microbiota and immune responses (40). These complex interactions may explain the significant gender-related interaction between the TyG index and the incidence of UC and CD.

The results of the non-linear analysis revealed a significant non-linear relationship between the TyG index and the risk of UC, characterized by an initial steep increase followed by a plateau. Although the non-linear relationship between the TyG index and CD risk did not reach statistical significance, graphical analysis suggested a trend of a gradual increase at first, followed by a sharper rise. These findings indicate that the impact of the TyG index on the risk of IBD may involve a threshold effect, where the risk becomes more pronounced within certain ranges of the TyG index. The identification of this non-linear relationship holds important clinical implications, as it suggests that the complex non-linearity should be taken into account when using the TyG index to assess IBD risk. This approach could facilitate more accurate identification of high-risk individuals and provide a foundation for developing personalized preventive strategies.

Research has demonstrated that higher TyG index levels are associated with an increased incidence of intestinal obstruction, fistula complications, and IBD-related surgeries in CD patients. This may result from the combined effects of exacerbated chronic inflammation, heightened oxidative stress, and accelerated fibrosis. Under conditions of insulin resistance, the secretion of pro-inflammatory cytokines, such as TNF-α and IL-6, is elevated, which may worsen chronic intestinal inflammation (41). Simultaneously, oxidative stress induced by hyperglycemia and hyperlipidemia may impair intestinal epithelial cells, disrupting the intestinal barrier function (42). Additionally, overexpression of insulin-like growth factor-1 (IGF-1) under insulin-resistant conditions may contribute to intestinal fibrosis (43). Therefore, in clinical practice, regular assessment of the metabolic status of IBD patients, including calculation of the TyG index, encouraging patients to improve their metabolic health through diet and exercise, and developing individualized treatment plans based on their metabolic status, may help prevent complications, reduce the need for surgery, and improve overall prognosis. Nevertheless, further prospective studies are required to validate these findings and to explore the specific effects of interventions targeting metabolic abnormalities in the management of IBD.

The primary strength of this study lies in its use of data from the UK Biobank, a large-scale, high-quality population-based cohort, which provides a substantial sample size and long follow-up period, offering robust statistical support for the findings. We employed a variety of statistical methods, including Cox regression, stratified analysis, interaction analysis, mediation analysis, and non-linear analysis, to comprehensively explore the relationship between the TyG index and IBD, as well as its related complications, from multiple perspectives. However, this study also has several limitations. As an observational study, although we identified a significant association between the TyG index and IBD risk, we are unable to establish a causal relationship between the two. Despite our efforts to adjust for multiple potential confounders, residual confounding factors such as unmeasured dietary patterns, environmental exposures, and genetic predispositions could still influence the observed association. The bidirectional relationship between metabolic dysregulation and inflammation also complicates causal inference in this context. Additionally, the UK Biobank primarily includes individuals of European descent, which limits the generalizability of the findings to other racial or ethnic groups. Finally, this study only considered the baseline TyG index, and thus did not capture the potential impact of temporal changes in the TyG index on IBD risk.

Based on the findings and limitations of this study, future research directions should include: conducting prospective cohort studies or randomized controlled trials to further validate the causal relationship between the TyG index and IBD risk; investigating the molecular and cellular mechanisms underlying the influence of the TyG index on IBD risk, particularly focusing on identified potential mediators such as CRP and inflammatory cell counts; assessing whether interventions that improve metabolic status (lowering the TyG index) can reduce IBD risk or improve disease outcomes; and evaluating the clinical utility of TyG index in IBD management through: (1) integration into risk prediction models for early IBD screening in high-risk populations, (2) use as a stratification tool for personalized treatment approaches, and (3) potential adoption as a monitoring parameter to assess treatment efficacy. These applications should be validated across diverse racial and ethnic populations, with longitudinal assessments capturing dynamic TyG changes. Implementation studies examining the feasibility and cost-effectiveness of incorporating TyG assessment into routine clinical practice would translate these findings into practical clinical protocols while maintaining the advantages of this simple, cost-effective biomarker derived from standard laboratory tests.

## 5 Conclusion

In summary, this study is the first to confirm a significant association between the TyG index and the risk of IBD in a large population. It also highlights the complexity and potential mechanisms underlying this relationship. These findings offer new insights into the pathogenesis of IBD, underscoring the importance of metabolic health in its prevention and management. Future research should further explore the causal relationship and clinical relevance of these findings, providing a scientific basis for the prevention, early diagnosis, and development of personalized treatment strategies for IBD.

## Data Availability

The raw data supporting the conclusions of this article will be made available by the authors, without undue reservation.
